# Application of Melt-Blown Poly(lactic acid) Fibres in Self-Reinforced Composites

**DOI:** 10.3390/polym10070766

**Published:** 2018-07-12

**Authors:** Dániel Vadas, Dávid Kmetykó, György Marosi, Katalin Bocz

**Affiliations:** Department of Organic Chemistry and Technology, Budapest University of Technology and Economics, Budafoki út 8, H-1111 Budapest, Hungary; vadas.daniel@mail.bme.hu (D.V.); kmetykod@gmail.com (D.K.); kbocz@mail.bme.hu (K.B.)

**Keywords:** poly(lactic acid), melt-blowing, nonwoven mat, self-reinforcement, thermal annealing, polymer composite

## Abstract

The aim of our research was to produce poly(lactic acid) (PLA) fibres with diameters in the micrometer size range, serving as the reinforcing phase in self-reinforced (SR) PLA composites. Nonwoven PLA mats were manufactured by solvent-free melt-blowing technology. Three types of PLA differing in d-lactide content were processed with a productivity as high as 36 g/h. The crystallinity of the PLA microfibres was enhanced by thermal annealing. A 2–3-fold increase in the degree of crystallinity was obtained, as measured by differential scanning calorimetry (DSC). Fibre diameters between 2–14 µm were revealed by scanning electron microscopy (SEM). Static tensile tests were performed on the nonwoven mats, showing the reduced moduli of the annealed fibres due the amorphous relaxation. The PLA mats were processed via the hot compaction technique and formed into SR–PLA composites. The morphological and mechanical properties of the obtained microstructural composites were comprehensively studied. Composites prepared from annealed, thermally more stable PLA nonwoven mats showed superior mechanical properties; the tensile strength improved by 47% due to the higher residual fibre content.

## 1. Introduction

As the conventional linear economic model has begun to shift towards a more sustainable circular economy—even at a moderate pace—more and more emphasis has been placed on the development of renewable and/or biodegradable polymers (collectively known as biopolymers) within the plastics industry. Compared to the rest of the plastics industry, the biopolymers market is expanding at an increasing speed [1]. Various types of natural polymers (cellulose derivatives, lignin, chitosan, pectin, alginate, polyhydroxyalkanoates, pullulan) and synthetic biopolymers (poly(glycolic acid), poly(lactic acid), poly(vinyl alcohol), polybutylene succinate, etc.) have been investigated over the last two decades [2]. Among the aimed uses are medical, packaging, and many industrial applications, especially in the form of biocomposites or nanobiocomposites [3,4,5,6].

The most intensively studied biopolymer, polylactic acid (PLA), has a market with a compound annual growth rate (CAGR) of 19.5%, which is expected to reach $5.2 billion by 2020 and $6.5 billion by 2025 [7,8]. The main advantage of PLA is that it can be processed using the conventional methods of the plastics industry (extrusion, injection molding, thermoforming, fibre drawing, etc.) [9]. Various products can be produced using this biopolymer, such as inter alia, blow-molded bottles, injection-molded cups, spoons and forks [10]. Nevertheless, in order to use PLA as a raw material for durable applications, it is necessary to increase its low impact and heat resistance. Researchers have recently demonstrated that with self-reinforcement (SR), a special type of composite production, the impact resistance of PLA can be improved [11,12]. In addition, since the reinforcement and matrix material of an SR–PLA product are both composed of PLA grades, the article remains fully biodegradable. This concept fits well into a sustainable, circular economic model, so lately, there has been increased scientific interest in self-reinforced biocomposites.

Jia et al. [13] combined oriented crystalline PLA fibres with amorphous PLA films with significantly different melting points in order to widen the processing window which exceeded 30 °C. With a fibre content of 22% (applying unidirectional orientation of the fibres), SR–PLA composites with a modulus of 3.29 GPa and tensile strength of 48 MPa were produced. Thus, the modulus increased by 140% and the tensile strength by 13% compared to the matrix material. It is worth mentioning that with a bidirectional orientation of the fibres, the modulus increased by only 74% and the tensile strength decreased by 65%.

Somord et al. [14] produced SR–PLA composites via hot compaction of PLA fibres manufactured by electrospinning. The PLA solution was prepared with a mixture of dichloromethane and dimethylformamide (7:3), and the fibre formation was carried out using 20 kV acceleration voltage and 18 cm collector distance. Fibre mats of 0.8 g were produced within a 2.5 h period, which equals a productivity rate of 0.32 g/h. The crystallinity of the produced fibres was 16% based on differential scanning calorimetry (DSC) measurements. After removing the moisture content of the fibres with ethanol, composite sheets with dimensions of 30 mm × 30 mm × 150 μm were pressed at 165 °C and 6 MPa, by varying the compression time from 10 to 60 s. The tensile strength and modulus of the composites (at 20 s compaction time: σ_y_ = 77.5 MPa, *E* = 3.2 GPa) improved compared to the properties of the isotropic PLA film (σ_y_ = 49.9 MPa, *E* = 2.8 GPa). Kriel et al. [15] prepared core-sheath PLA fibres composed of a semicrystalline core and amorphous sheat by coaxial electrospinning. The bicomponent fibre structure ensured a wide processing window for SR composite preparation. Thermal treatment of the electrospun fibres was found to be essential to increase crystallinity and mechanical strength. Nevertheless, the low productivity of electrospinning and the involved organic solvents make this method hardly scalable; the application areas of electrospinning are limited to small size products with high added value [16,17,18,19,20].

From a feasibility point of view, conventional fibre production techniques are more advantageous, because production can be accomplished at significantly higher speeds and in higher quantities. Melt-blowing is one of the most cost-effective and versatile commercially available processes to produce microfibrous products. The definition of this technique is ‘a one-step process in which high-velocity air blows molten thermoplastic polymer from an extruder die tip onto a conveyor to form a fine fibered web’ [21]. Melt-blowing technology has also been used to manufacture PLA nonwoven mats, targeting innovative applications such as special tissue scaffolds [22] and filters [23]. However, the utilization of melt-blown PLA microfibers to form SR composites has been barely studied in the literature. Recently, melt-spun core-sheat PLA fibres with a melt processing window as wide as 40 °C were transformed into SR–PLA composites via hot-pressing by Liu et al. [24]. The hot-pressing temperature was found to have a noticeable effect on the composites’ morphological and mechanical properties.

The present study demonstrates the manufacturing method of one-component of microfibrous PLA mats by the solvent-free melt-blowing technique, focusing on the effect of the d-lactide content and thermal annealing on the morphological, thermal and mechanical properties of the produced PLA fibres. The obtained nonwoven mats were further processed by hot compaction to form SR–PLA composites, the corresponding properties of which were investigated as well.

## 2. Materials and Methods

### 2.1. Materials

As the stereoisomeric purity of PLA significantly influences its mechanical and thermal properties [25], PLA grades possessing comparable rheological properties (MFIs), but differing in d-lactide content, were selected for fibre production. 3052D, 3001D and 3100HP Ingeo^TM^ Biopolymer PLA produced by NatureWorks LLC (Minnetonka, MN, USA) were chosen. Some of the most relevant properties of the PLA types used are summarized in Table 1.

### 2.2. Melt-Blowing

PLA fibres were produced by melt-blowing from raw materials previously dried for at least 8 h at 85 °C. A Quick Extruder QE TS16 02/2016A type twin-screw pharmaceutical extruder (QUICK 2000 Ltd., Tiszavasvári, Hungary) was used with an l/d ratio of 25. The four heating zones of the extruder were heated to 200 °C, the die temperature was 170 °C and the screw speed was set to 15 rpm. A specially designed adapter was attached to the extruder die to allow the formation of sufficiently fine fibres and an appropriate flow of hot air, i.e., the melt-blowing process. The die had 330 μm diameter holes next to each other, and the compressed air with an overpressure of 1 bar was heated by an AHP-7562 type device supplied by OMEGA Engineering INC, Stamford, CT, USA. The air temperature was set to 300 °C. For the collection of PLA microfibres, a hemispherical sieve made of metal mesh placed at a distance of 25 cm from the die was used. By means of melt-blowing, 0.6 to 0.7 g of fabric was produced per minute, corresponding to a productivity rate of 36 g/h. This is 110–130 times higher than the productivity of the electrospinning method used by Somord et al. [14].

### 2.3. Thermal Annealing

The produced melt-blown webs were largely amorphous due to rapid cooling, and since the crystalline fraction plays a key role in the production of composites, thermal annealing experiments were carried out above the glass transition temperature (*T*_g_). Samples of the microfibrous mats were placed into an 85 °C oven for 2 h. In the first hour, samples were taken every 15 min and then after 120 min, from which the effect of post-crystallization was investigated by DSC.

### 2.4. Composite Preparation

SR–PLA composites were prepared from annealed and non-annealed nonwoven PLA mats of the 3100HP type PLA, which proved to be the most promising material. In the case of non-annealed mats, the moisture was removed by drying for 1 h at 50 °C to avoid hydrolysis during hot compaction. From the webs, 26.6 × 26.6 mm^2^ squares were cut, which were layered into a square mould with dimensions of 30 × 30 × 0.4 mm^3^. The mould was placed between two metal sheets coated with polytetrafluoroethylene (PTFE) foil. The hot compression process was carried out with a Collin GmbH Teach-Line Platen Press (Ebersberg, Germany) 200E hydraulic press at 165 °C and 60 bars for 4 different durations (10, 20, 30 and 60 s) for the annealed mats. Non-annealed fibres were also processed at 160 °C, 60 bars, for 20 s, using the same apparatus. After the hot compression was completed, the mould was cooled to room temperature via cooling in water for 7 min under pressure.

### 2.5. Scanning Electron Microscopy (SEM)

A JEOL JSM-6380LA type scanning electron microscope (Tokyo, Japan) was used to examine the morphology of the fibres and the microstructure of the composites. The SEM images were taken with an accelerating voltage of 15 keV. All the samples were coated with gold–palladium alloy before examination in order to prevent charge build-up on their surfaces.

### 2.6. Differential Scanning Calorimetry

The thermal properties of the fibres were studied using a TA Instruments Q2000 type calorimeter (New Castle, DE, USA). DSC measurements were carried out at a heating rate of 10 °C/min under 50 mL/min nitrogen gas flow, covering a temperature range of 30–200 °C. About 4–9 mg of sample was measured in each test using 26.4 mg aluminum pans. The degree of crystallinity (χ) of the samples was calculated according to Equation (1):

(1) $$X=\frac{\Delta H_{m}-\Delta H_{cc}}{\Delta H_{f}}*100\;(\%)$$

where Δ*H_m_* indicates the melting enthalpy, Δ*H_cc_* is the cold crystallization enthalpy and Δ*H_f_* is the melting enthalpy of the 100% crystalline PLA equal to 93 J/g [26].

### 2.7. Tensile Testing

Static tensile tests were performed on the annealed and non-annealed microfibrous mats, and also on the SR composites. Samples (7.5 mm × 30 mm) of the microfibrous mats and specimens (3 mm × 30 mm) of the SR composites were cut out and tested on a ZWICK Z005 universal testing machine (Zwick GmbH & Co., KG, Ulm, Germany). For the samples of the mats, a 20 N load cell was used, the initial grip separation was 11 mm, and the crosshead speed was set to 5 mm/min. Regarding the composite specimens, the measurements were performed on a 5 kN load cell with an initial grip separation of 10 mm and crosshead speed of 1 mm/min.

## 3. Results and Discussion

### 3.1. Fibre Morphology

**Figure 1:**
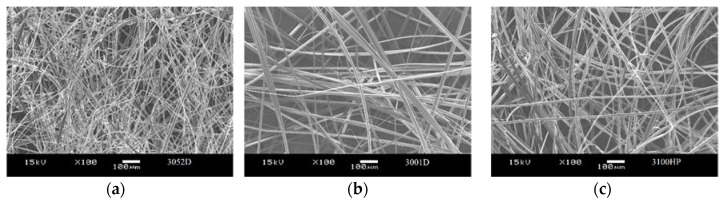

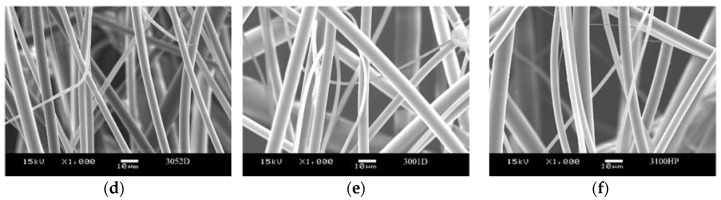
SEM images of the melt-blown PLA nonwoven mats: (**a**,**d**) 3052D; (**b**,**e**) 3001D; (**c**,**f**) 3100HP. Magnification: ×100, ×1000.

**Figure 2:**
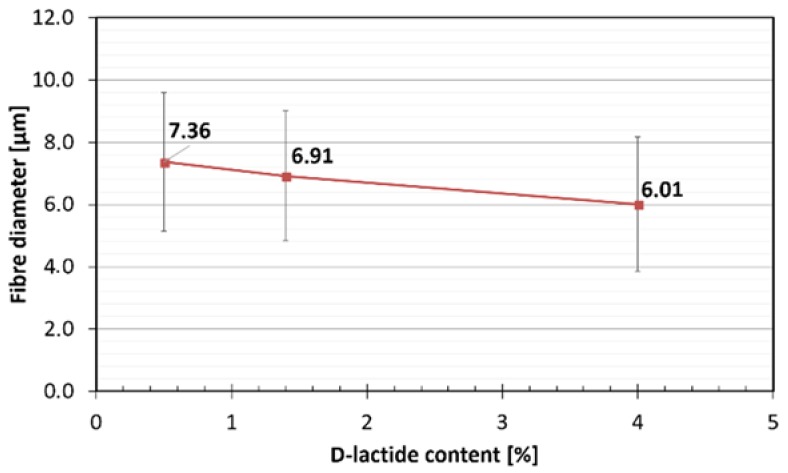
Diameters of the melt-blown PLA fibres.

**Figure 3:**
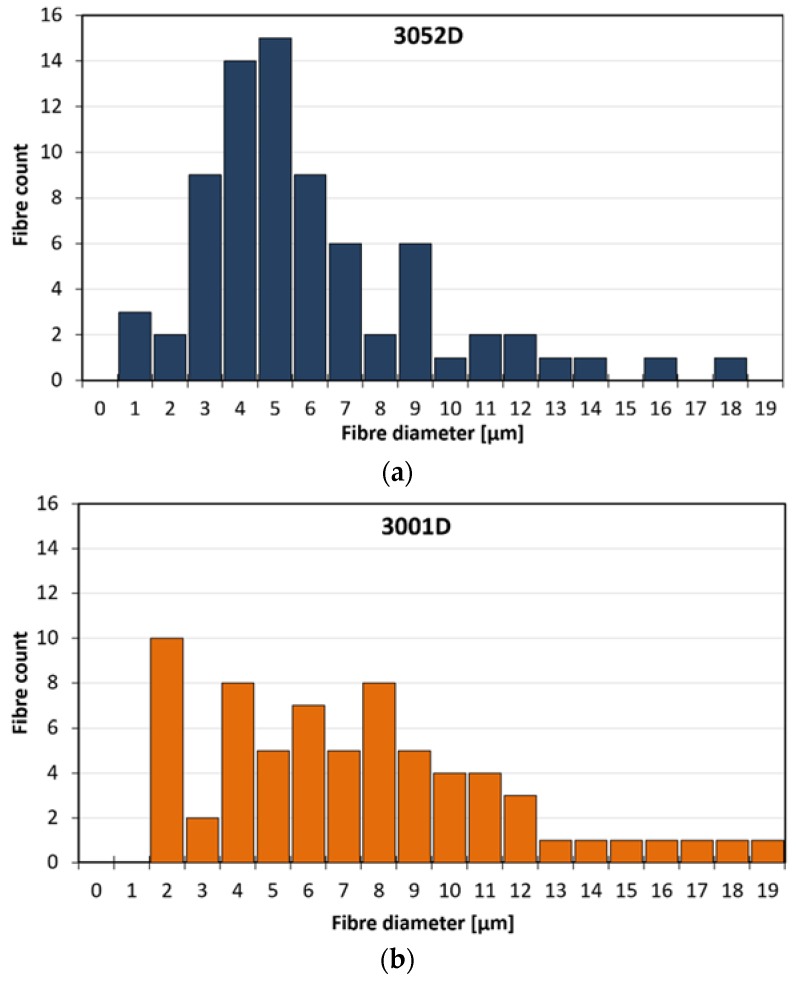

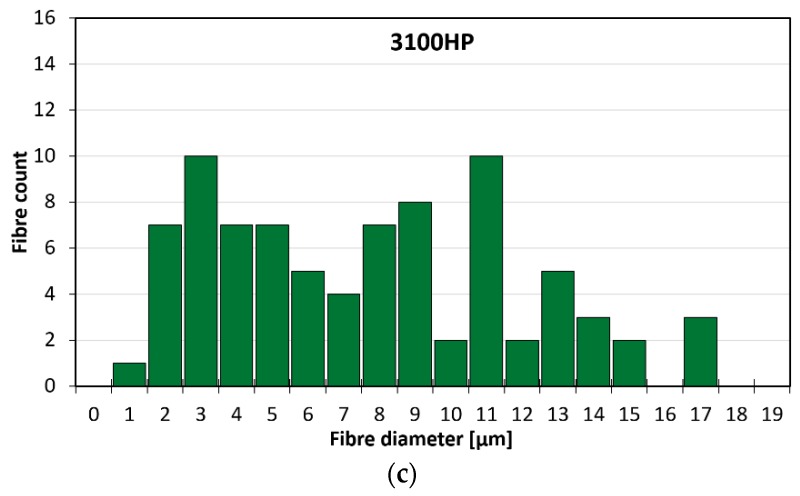
Diameter distribution of fibres obtained from 3052D (**a**), 3001D (**b**) and 3100HP (**c**) grade PLA.

The morphologies of the nonwoven mats and the fibre diameters were investigated by SEM analysis. As it can be observed in the images with magnifications of ×100 and ×1000 (Figure 1), PLA fibres are randomly stacked in several layers, showing longitudinal bonding in numerous locations. The average fibre diameters and the fibre diameter distributions of the prepared three types of PLA nonwoven mats are shown in Figure 2 and Figure 3, respectively. The diameter of the melt-blown fibres varied between 2 and 14 μm for each type of PLA used, which is greater than the diameters of fibres produced by electrospinning in the literature [14]. In Figure 2, a decreasing tendency of fibre diameters can be observed as a function of PLA’s d-lactide content, but the difference is not significant. The measured fibre diameter values (at least 70 fibres were measured from each type of PLA nonwoven mat) were statistically tested, and we were able to reject the null hypothesis that the slope of the regression line for the fibre diameters of increasing d-lactide contents is zero (H_0_: β_1_ = 0) with a probability value of *p* = 0.045 which is close to the generally used significance level of α = 0.05.

### 3.2. Thermal Properties—Crystallinity

**Figure 4:**
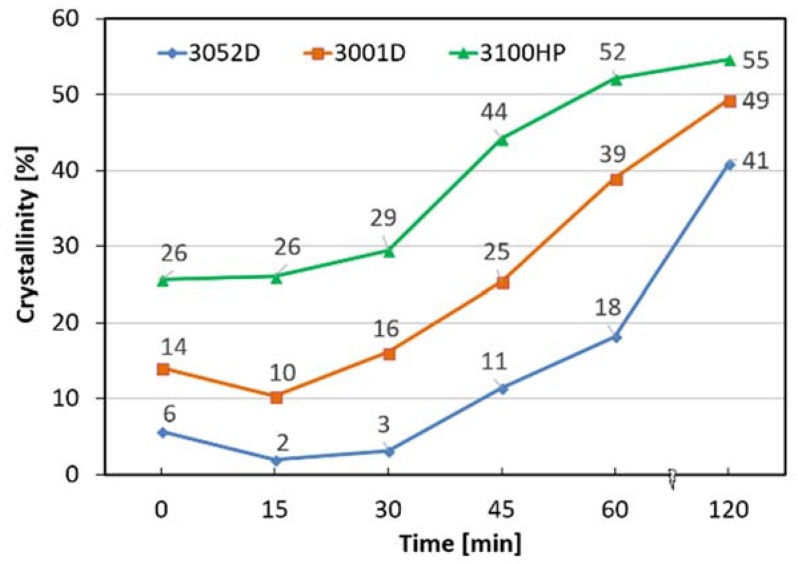
Crystallinity of PLA fibres as a function of annealing time.

DSC analyses were carried out to investigate the thermal properties and crystallinity of the annealed and non-treated fibres. As can be seen in Figure 4, depending on the d-lactide content of the used PLA type, a two to seven-fold increase in crystallinity was reached after two hours of annealing. Nevertheless, it can be observed that this procedure erases the thermal history of the polymer and creates a new structure. During the melt-blowing process, orientation and alignment of the PLA macromolecules in the direction of the fiber axis occurred, initiating crystallization and ordering of the amorphous region at the same time. Annealing at 85 °C, above the glass transition temperature of PLA (~60–66 °C), enhances segmental mobility and the oriented polymer chains try to return to their thermodynamically more stable form. These phenomena explain the decrease in crystallinity in the first period (15 min) of thermal annealing. For the 3100HP type PLA, the two processes compensate each other so that total crystallinity is not reduced. Then, during cold crystallization, the amorphous parts of the macromolecules are reorganized, but the longitudinal axis of the fibre is not a preferred direction anymore, and the crystallinity shows an increasing tendency as a function of the annealing time for all polymer types.

**Figure 5:**
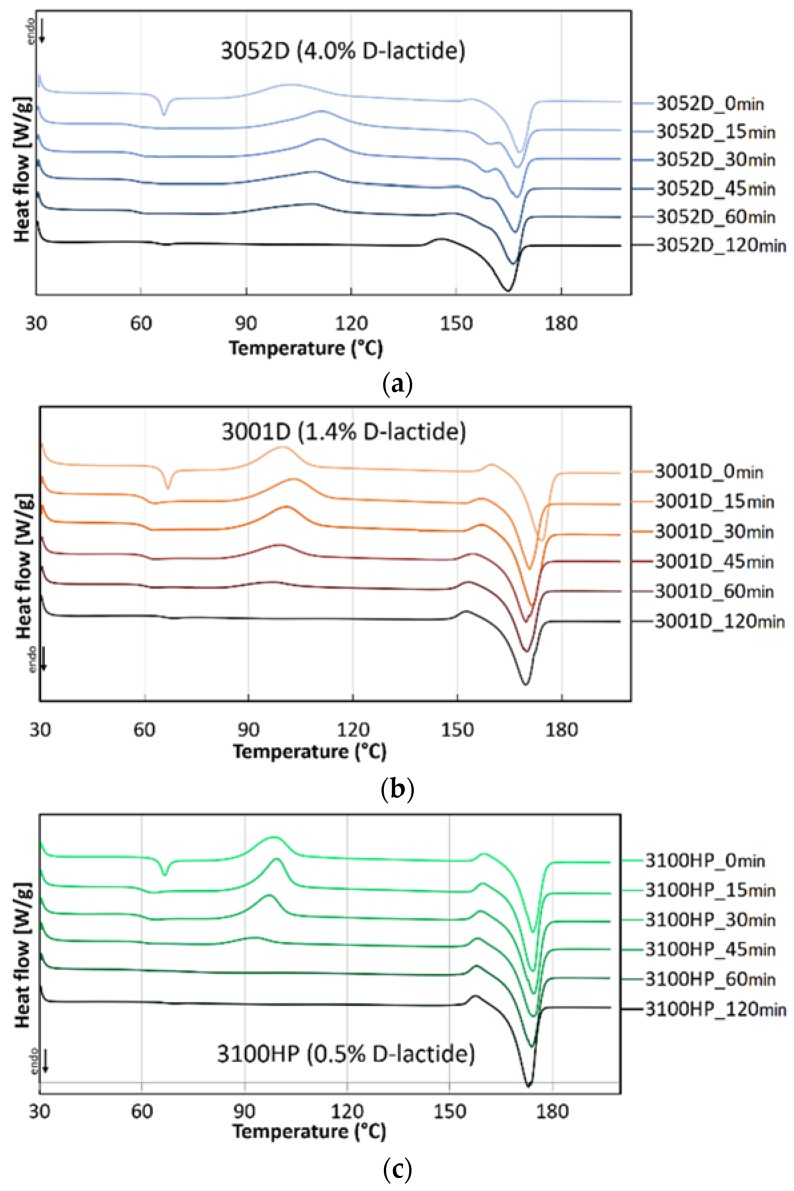
Thermograms of 3052D (**a**), 3001D (**b**) and 3100HP (**c**) type PLA annealed for 0–120 min.

The thermal transitions of the PLA fibres with differing d-lactide-contents can also be observed on the corresponding DSC curves (Figure 5). In the case of non-annealed (0 min) samples, the *T*_g_ is observed around 66 °C. On the curves of the annealed fibres, this phenomenon is marked by a much smaller thermal effect as the frozen-in strains induced during the melt-blowing process are eliminated in 15 min. As the crystallinity increases with the annealing time, the exothermic peak of cold crystallization decreases, and after 30 min of annealing, it is barely noticeable. For the samples annealed for 2 h, this heat transition is not visible at all, indicating that the fibres have reached their maximum crystallinity.

It can be observed that after 15 min of thermal treatment, the cold crystallization peak temperature significantly increased. The shift of cold crystallization exotherm to the lower temperature of the non-treated fibres is attributed to the strain-induced nucleation enhanced crystallization of the stretched amorphous phase. As there is no orientation in the annealed fibres, the ordering of the macromolecules requires extra energy (higher temperature). At higher d-lactide contents (Figure 5a), this effect causes a significant difference, but it is barely noticeable for 3100HP (Figure 5c), as in the latter case, crystallization is facilitated by the presence of a high amount of pre-existing crystals (χ = 26%). After increasing the heat treatment time, the cold crystallization peak temperatures showed a slightly decreasing tendency in all cases, which is also due to the increasing crystallinity.

For the annealed 3052D (15–60 min) PLA fibres (Figure 5a), a double endothermic crystalline melting peak can be observed, which means that both crystalline forms of PLA (the less ordered α′ and the more ordered α crystalline forms) are present. The smaller peak at 159 °C shows the melting of the α′ form and the recrystallization of the α crystal form; the larger peak refers to the melting of the α form. The 3052D type PLA contains the highest amount of d-lactide (4.0%), which decreases the regularity of the macromolecules so that α′ crystalline formation can occur. It can be seen that after 120 min, these less ordered crystalline structures also transform into a thermally more stable α crystalline form. This curve as well as the ones of 3001D and 3100HP PLA types show a smaller exothermic peak prior to crystalline melting. From this we conclude that during the heat treatment, α′ is formed, and this exothermic peak indicates solid phase transformation into the more stable α form occurring in the DSC apparatus [27]. The crystalline melting peak temperature increases with a decreasing d-lactide content (3052D: 167 °C, 3001D: 172 °C, 3100HP: 175 °C), this effect is also due to the higher macromolecular regularity of the optically pure PLA types.

### 3.3. Mechanical Properties of the Microfibrous Mats

**Figure 6:**
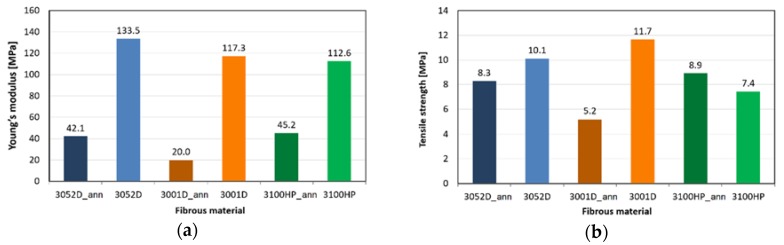
Young’s modulus (**a**) and tensile strength (**b**) of annealed (ann) and non-annealed PLA mats.

The results of the tensile tests are shown in Figure 6. The mechanical characteristics of the melt-blown microfibrous mats are comparable with the modulus and strength of electrospun PLA nonwoven mats, as found in the literature [28]. It can be noticed that the Young’s moduli of the annealed mats are much smaller than that of the non-annealed mats obtained from the same material. This phenomenon can be explained by macromolecular processes occuring during heat treatment. During thermal treatment, the amorphous orientation formed in the PLA fibres is relaxed, so the modulus is also reduced [29]. Regarding the tensile strength—except for the 3100HP type—the non-annealed mats also outperform the annealed ones. As the tensile strength is more influenced by the orientation of the crystalline part, the differences between the values are smaller.

### 3.4. Mechanical Properties of SR Composites

**Figure 7:**
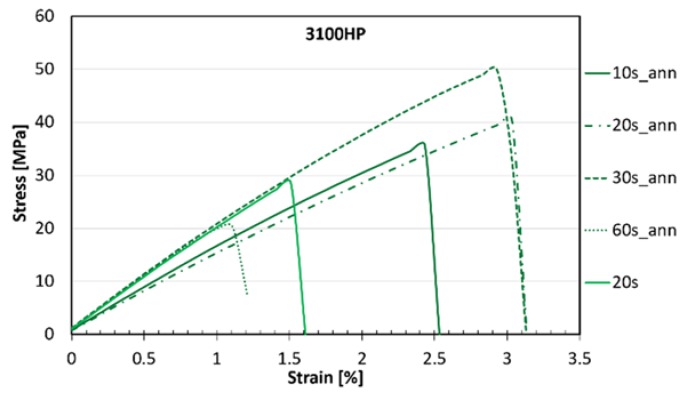
Stress–strain curves of SR–PLA composites.

**Figure 8:**
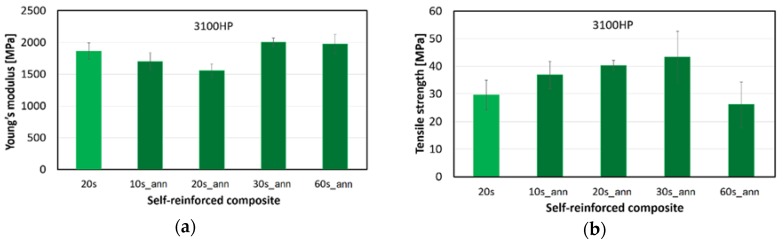
Young’s modulus (**a**) and tensile strength (**b**) of SR composites made of annealed (ann) and non-annealed PLA mats, indicating the hot compaction time (10–60 s).

Based on the DSC measurements, crystallinity data and mechanical properties of the melt-blown PLA nonwoven mats, the 3100HP type PLA was selected for SR composite preparation. The typical stress–strain curves of the obtained composites can be seen in Figure 7, while the modulus and tensile strength values are shown in Figure 8.

In contrast to the tensile test results of the nonwoven mats, annealed fibres compacted for 30 s (30s_ann) showed slight improvements in modulus when compared to SR–PLA specimens composed of non-treated mats (20 s). The significant effect of thermal treatment of the fibrous mats was also evidenced by the obtained 47% increase in tensile strength, reaching 43 ± 9 MPa in the case of the 30s_ann composite. These favorable mechanical properties are connected to the high crystallinity achievable in the case of the low (0.5%) d-lactide containing PLA type, providing suitable thermal resistance for processing by hot compaction. However, 60 s of hot compression resulted in noticeable deterioration of elongation at break (Figure 7) and tensile strength (Figure 8a) values of the SR-PLA composite, likely due to the partial melting and fusion of the microfibers and also to the physical ageing that occurs during longer processing times.

### 3.5. Morphology of the SR–PLA Composites

**Figure 9:**
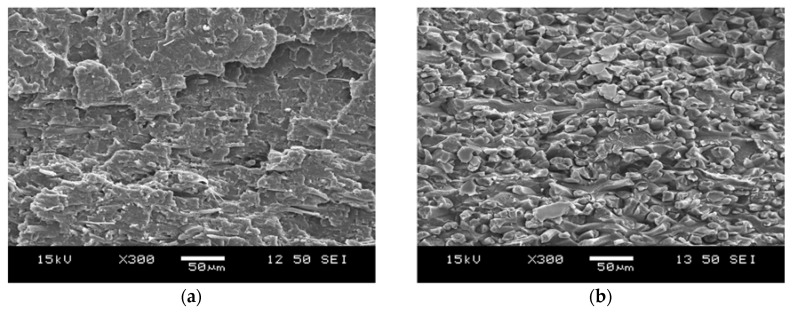
SEM images of self-reinforcement (SR)–PLA composites made from non-annealed (**a**) and annealed (**b**) fibres with 0.5% d-lactide content (3100HP). Magnification: 300×.

The fracture surfaces of the SR–PLA specimens were analyzed by SEM. Based on the SEM micrographs presented in Figure 9, conclusions regarding the consistency, fibre orientation and failure mechanism of the composites were drawn. Despite the 5 °C lower processing temperature but identical hot compaction time (20 s), a significantly lower amount of reinforcing fibre was observed in the fracture surface of the composite made from non-treated PLA mats (Figure 9a), while fibres that underwent thermal annealing mostly remained intact during processing (Figure 9b). The more than two-fold increase in crystallinity resulted in higher thermal resistance of the microfibres, and thus, lower sensitivity to the high compression temperature.

In the SEM images, three different failure modes can be observed, namely, fibre pullout, fibre/matrix debonding and brittle failure of fibres. Composites made from highly amorphous fibres broke with plastic deformation, but specimens with higher crystallinity suffered brittle fibre failure. In the case of the SR composite composed of thermally annealed microfibers, only a suitable fraction (surface) of the reinforcing fibres were molten during processing, forming the matrix phase, and thus well-consolidated composites could be obtained. In this case, self-reinforcement was successfully implemented.

## 4. Conclusions

In this work, PLA microfibrous nonwoven mats, serving as precursors for self-reinforced composite preparation, were prepared by melt-blowing technology. Fibres with diameters ranging from 2–14 µm were obtained with a productivity rate of 36 g/h from three types of PLA grades differing in d-lactide content. The crystalline fractions of the obtained fibres were significantly increased by thermal annealing at 85 °C for 2 h with the aim of improving their thermal resistance. The heat treatment induced, however, relaxation of the molecular orientation in the fibres, and thus a decreased modulus values was measured for the annealed fibres. Nevertheless, self-reinforced composites with improved mechanical performance and adequate morphology could only be obtained from thermally pre-treated fibres. The improved thermal resistance of the highly crystalline PLA microfibres proved to be of key importance regarding the ability of partial melting, i.e., matrix formation, and to obtain adequate consolidation quality by hot compaction.

## Figures and Tables

**Table 1:** Properties of the selected polylactic acid (PLA) types.

Type	3052D	3001D	3100HP
Density (g/cm^3^)	1.24	1.24	1.24
MFI (g/10 perc) (210 °C, 2.16 kg)	14	22	24
d-lactide content (%)	4.0	1.4	0.5
Crystalline melt temperature (*T*_m_) (°C)	145–160	160–175 *	165–180 *
Glass transition temperature (*T*_g_) (°C)	55–60	55–60 *	55–60 *

* Estimated based on differential scanning calorimetry (DSC) measurements.
